# Social media use and job choices: the mediating roles of work values and self-efficacy

**DOI:** 10.3389/fpsyg.2025.1485663

**Published:** 2025-02-13

**Authors:** Fei Li, MinXuan Shi, Ran Feng

**Affiliations:** ^1^School of Humanities, Anhui Polytechnic University, Wuhu, Anhui, China; ^2^School of Public Administration, Hohai University, Nanjing, Jiangsu, China

**Keywords:** social media, college students, job choices, work values, self-efficacy

## Abstract

This study examines the association between social media use and college students’ employment choices, focusing on the roles of work values and self-efficacy as mediating factors. A survey of 254 college students in Anhui Province showed that social media users tended to have more flexible and diverse employment options than non-users. Regression analyses demonstrated a positive association between social media use and employment decisions, with work values and self-efficacy identified as mediating factors in this relationship. Notably, self-efficacy was found to enhance the association between work values and students’ employment choices. These findings suggest that fostering students’ self-efficacy enhances the positive impact of work values, emphasizing the need for career guidance to encourage appropriate use of social media while avoiding over-reliance. This study provides valuable insights into promoting full employment among college students by considering the role of social media in shaping their career decisions.

## 1 Introduction

In recent years, the rapid advancement of the Internet and mobile communication technologies has positioned social media as a central component of college students’ daily lives. Social media has not only transformed communication practices but has also significantly impacted the processes of information acquisition and processing. Numerous studies have demonstrated that social media exerts a substantial impact on individuals’ career development and employment decisions (Ostic et al., 2021; Nikitkov and Sainty, 2014). As a primary user group of social media, the work values and career choices of 90s and 00s college students significantly relate to these platforms. In today’s job market, college students increasingly rely on social media to gather background information on potential employers, enabling them to effectively align recruitment opportunities with their personal career goals and needs (Gershon, 2024). Therefore, social media functions not only as a channel for information dissemination but also as a tool for corporate impression management. This evolving employment landscape has prompted college students to carefully consider their performance and interactions on social platforms during their job search. Additionally, success stories and corporate culture representations on social media subtly shape college students’ work values. Work values encompass the intrinsic principles and expectations that individuals prioritize when selecting employment, including factors such as job stability, career advancement opportunities, compensation, and social recognition (Shi et al., 2023).

Sharing experiences related to career fulfillment and work-life balance on social media platforms may inspire college students to reassess traditional employment concepts and explore more diverse career paths. The concept of work and employment choice conflict refers to the dilemma individuals face when choosing between various career roles, given limited time and resources. Pursuing one career objective often necessitates the sacrifice of another, leading to conflict in decision-making (Ma et al., 2014). Today’s college students may be just entering the workforce or may be in the exploratory stage of their careers. While pursuing well-paying jobs and social recognition, they may forgo the opportunity to pursue a combination of work and interest, or even choose to abandon certain career paths (Lowe, 2021). Social learning theory states that individuals learn and form their own behavioral patterns by observing the behaviors, attitudes, and consequences of others (Bandura and Walters, 1977). This theory is especially applicable in a social media environment. College students form or adjust their work values and career choices by observing the life status and career development of professionally successful individuals on social media. In addition, the display of career information and the career dynamics of their peers on social media may lead them to value specific career attributes, such as creativity, flexibility, or high income (Ruparel et al., 2020; Jue et al., 2009). However, the direct financial benefits of pursuing a high-income position are obvious, but may be accompanied by a significant time and mental commitment, making it impossible for individuals to find a balance between work and personal life (Szromek and Wolniak, 2020). This imbalance leads to the rational consumption theory of employment choice, where individuals follow a rational balance of utility and cost in their employment behavior (Fischer et al., 2021). While the benefits of career development are often long-term and less immediately apparent, the associated costs-such as time, opportunity costs, and psychological stress-are immediate and visible. Furthermore, the distinct characteristics of social media use, coupled with specific economic and cultural contexts, are particularly pronounced in China. Through social media observation and learning, college students may experience “expectation pressure” in the job market, driven by societal definitions of “success, “which can result in skewed or biased employment choices (Xu, 2023). As a result, some university students opt to delay entry into the workforce, progress at a slower pace, or alter their career trajectories in pursuit of professions that align more closely with their interests and personal values (Lent and Brown, 2020).

As society progresses and living standards improve, the costs associated with pursuing career satisfaction and personal development have correspondingly increased. These costs extend beyond financial investments to include emotional commitment, the cultivation of professional values, and the development of personal skills and interests. Consequently, college students often struggle to balance their career aspirations with their personal lives, leading to a diminished preference for traditional career paths, particularly those that demand long working hours and high levels of dedication (Kossek et al., 2021).

## 2 Review of literature

### 2.1 Social media impact: a social learning theory perspective

In the digital age, social media has emerged as a powerful platform that fundamentally transforms how individuals learn, interact, and make decisions. Through the Social Learning Theory, we can comprehensively understand the profound impacts of social media on human behavior and societal dynamics. Social Learning Theory, developed by Bandura, emphasizes learning through the observation of others’ actions and their outcome s. This theory is centered around four core concepts: observational learning, imitation, self-efficacy, and reinforcement mechanisms (Rumjaun and Narod, 2020). Social media offers a dynamic and evolving platform for the application of Social Learning Theory, where users can acquire new skills, behaviors, and knowledge by watching content (e.g., videos, pictures, and posts) posted by others (Huang and Chang, 2020). With the global rise of social media, its relevance on learning, knowledge sharing, and business value creation has garnered significant attention. In recent years, researchers have analyzed how social media influences individual and organizational learning behaviors and performance from various perspectives. Hosen et al. (2021) examined the impact of individual motivation and social media on students’ knowledge sharing and learning outcomes, revealing that social media significantly enhances learning performance by fostering interaction and knowledge exchange among students. This finding underscores the pivotal role of social media platforms in facilitating observational learning and imitation, enabling students to observe and replicate the learning behaviors of others, thereby improving their own learning outcomes (Hosen et al., 2021). Ansari and Khan (2020) explored the role of social media in collaborative learning, noting that social media platforms provide students with an interactive and collaborative environment that facilitates knowledge sharing and co-creation. This is in line with the concept of collaborative learning in social learning theory, whereby by interacting with peers and teachers on social media, students are able to access different sources of knowledge and learning strategies to enhance the depth and breadth of their learning (Ansari and Khan, 2020). Zafar et al. (2021) analyzed the impact of social media celebrities’ posts and contextual interactions on impulse buying behavior in social commerce and found that social media celebrities’ recommendation and display behaviors are significantly related to consumers’ purchase decisions through observational learning and imitation. This suggests that by imitating influential individuals, users’ behaviors and decisions are strongly related to each other, further validating the application of social learning theory to consumer behavior (Zafar et al., 2021). Zhang et al. (2020) investigated how co-creation between customers and businesses through social media drives business value, adopting an organizational learning and social capital perspective. The study found that through social media platforms, customers and firms can exchange and share knowledge and information, facilitating organizational learning and social capital accumulation (Zhang et al., 2020). This is consistent with the social learning theory of observational and interactive learning, whereby by observing and mimicking customer needs and feedback, firms are able to continuously optimize their products and services to achieve business value. Finally, Sun and Zhang (2021) reviewed the various theories and models applied in social media addiction research and made suggestions for future research. The article points out that social learning theory is important in explaining social media addictive behavior. By observing the usage behavior of others, users may imitate and gradually develop a dependence on social media (Sun and Zhang, 2021). In summary, these studies collectively highlight the significant role of social media in knowledge sharing, collaborative learning, consumer behavior, and organizational learning through the Social Learning Theory. By enabling observation, imitation, and interaction, social media platforms enhance both individual and organizational learning. Future research could delve deeper into the social learning processes across various social media platforms and user groups, offering fresh insights and data to advance theory and practice.

### 2.2 Self-efficacy

Self-Efficacy refers to an individual’s belief in his or her ability to accomplish a specific task or cope with a specific situation. Developed by Albert Bandura in 1977, the concept emphasizes an individual’s self-confidence and ability to perform in the face of challenges and has quickly become an important part of social cognitive theory (Calicchio, 2023). In recent years, with the popularity of social media, researchers have begun to explore the impact of social media on self-efficacy, particularly in the areas of work, health information access, knowledge sharing, and consumer behavior. Pekkala and van Zoonen (2022) examined the relationship between work-related social media use and social media communication self-efficacy. The study found that social media use in work scenarios can contribute to employees’ productivity and career satisfaction by enhancing their social media communication self-efficacy (Pekkala and van Zoonen, 2022). This suggests that social media as a communication tool can enhance employees’ self-efficacy and communication skills in a professional setting, thereby improving job performance (Ashaye et al., 2023). Niu et al. (2021) explored the association between health literacy, social media use and self-efficacy with health information acquisition intentions. Through a cross-sectional survey of Chinese social media users, it was found that self-efficacy played a mediating role in the health information acquisition process. High levels of health literacy and frequency of social media use could further increase users’ intention to actively acquire health information by enhancing their self-efficacy (Niu et al., 2021). Alshahrani and Rasmussen Pennington (2020) investigated the role of self-efficacy and its sources in social media knowledge sharing. The study showed that social media platforms facilitate knowledge sharing, while users’ self-efficacy is significantly related to their willingness to engage in knowledge sharing on these platforms. Through the interaction and feedback mechanisms of social media, users can enhance their self-efficacy and then actively participate in knowledge sharing activities (Alshahrani and Rasmussen Pennington, 2020). Tajurahim et al. (2020) explored the effects of consumer education intensity, self-efficacy, personality traits and social media on consumer empowerment. The study found that self-efficacy played a key role in the impact of consumer education and social media use on consumer empowerment. Specifically, social media empowers consumers by increasing their self-efficacy and enhancing their self-confidence and independence in consumer decision-making (Tajurahim et al., 2020). Huang and Zhang (2020) examined the relationship between social media use and entrepreneurial intentions and explored the mediating role of self-efficacy. It was found that social media use can significantly increase an individual’s entrepreneurial intentions by enhancing their self-efficacy (Huang and Zhang, 2020). This suggests that social media provides a platform for potential entrepreneurs to access resources, network, and obtain feedback, thereby increasing their entrepreneurial self-efficacy and action.

### 2.3 Work values

In recent years, research on the work values of college students has been explored in depth in different contexts. Ling et al. (2021) examined the employment of Chinese college students during a major public health event and found that the impact of the outbreak on the job market led students to focus more on the stability and security of their employment (Ling et al., 2021). Davidovitch et al. (2023), on the other hand, focused on the autism spectrum college graduates’ employment choices and labor market integration, noting the importance of inclusivity and equity in work values (Davidovitch et al., 2023). Perna’s (2023) edited volume Understanding the Working College Student summarizes current research on working college students, highlighting the positive impact of work experience on college students’ career development that It also reflects students’ values in balancing academics and work (Perna, 2023). Jackson and Tomlinson’s (2020) study showed that career planning and initiative play an important role in enhancing higher education students’ perceptions of employment under uncertain labor market conditions, which reflects students’ positive attitudes toward their future careers and values of self-efficacy (Jackson and Tomlinson, 2020). McCormick et al. (2023) explored the impact of working during college on student engagement and educational outcomes, further revealing how working college students enhance their skills and employability through work practice, leading to pragmatic and practical experience-focused work values (McCormick et al., 2023). Taken together, these studies reveal the diversity and complexity of university students’ work values in different contexts, emphasizing the centrality of stability, security, fairness, self-efficacy and practical experience in them.

## 3 Research hypotheses

In this study, we propose a series of hypotheses to explore the potential associations between social media use and college students’ job choices. Drawing from existing literature, we explore the complex interrelationships among social media usage, work values, self-efficacy, and career decision-making processes.

### 3.1 Social media use and college students’ work values

Work values are defined as the beliefs, attitudes, and priorities that guide individuals when making career choices, such as job stability, career advancement, and work-life balance (Munn, 2013). Previous research has shown that social media can be associated with college students’ career expectations and job choices by providing information, fostering social networks, and enhancing personal identity and sense of belonging (Gati and Kulcsár, 2021). Studies indicate that social media platforms may be positively related to students’ work values by offering access to relevant information, opportunities for networking, and even shaping their understanding of career goals (Abessolo et al., 2021; Waworuntu et al., 2022; Heidari et al., 2023; Shujaat et al., 2021; Nayak et al., 2020). Based on these findings, we propose that Social media use is positively associated with college students’ work values.

*Hypothesis 1*: Social media use is positively associated with college students' work values.

### 3.2 The mediating effect of work values

Work values are crucial drivers of an individual’s professional behavior and decision-making process (Sulistiobudi and Hutabarat, 2022; Ma et al., 2021; Wang and Li, 2022). Research shows that work values are associated with how college students assess different career opportunities, including how they prioritize factors such as job stability, career development opportunities, and work-life balance. Previous studies have demonstrated that work values are related to job choices by guiding individuals in evaluating available job opportunities (Perna, 2023; Huang et al., 2020). Therefore, it is hypothesized that work values mediate the association between social media use and job choices. Specifically, social media usage may be associated with work values, which in turn are associated with students’ final job choices.

*Hypothesis 2*: College students’ work values mediate the positive association between social media use and their job choices.

### 3.3 The mediating effect of self-efficacy

Self-efficacy refers to an individual’s belief in their ability to accomplish tasks and achieve goals. High self-efficacy is linked to increased confidence and better decision-making skills, particularly in career-related contexts. Research suggests that self-efficacy may play a role in shaping job choices, especially by reinforcing work values. When students have high self-efficacy, they are more likely to pursue careers aligned with their values, such as seeking out positions that offer job satisfaction, stability, and opportunities for growth (Wei et al., 2020). Studies have demonstrated that self-efficacy serves as a mediator between work values and job choices, as it helps students make more informed and confident decisions. Therefore, it is proposed that self-efficacy mediates the association between college students’ work values and their job choices.

*Hypothesis 3*: Self-efficacy mediates the positive association between college students' work values and their job choices.

### 3.4 The interaction between work values and self-efficacy

There is a complex interaction between work values and self-efficacy, with each being associated with the other. Research has shown that work values can enhance self-efficacy by influencing how students approach their career decisions, thereby boosting their self-efficacy and career confidence. Similarly, self-efficacy can strengthen the importance of work values by improving students’ perception of their career potential. This mutually reinforcing relationship may be amplified by social media, which serves as a platform for acquiring career information, establishing professional networks, and receiving social support. Therefore, the interaction between work values and self-efficacy may act as a mediator in the association between social media use and job choices, helping students make better career decisions.

*Hypothesis 4*: The interaction of work values and self-efficacy mediates the positive association between social media use and college students’ job choices.

### 3.5 Social media use and college students’ job choices

Social media platforms play an increasingly significant role in the career paths of college students. They serve not only as communication tools but also as key resources for career planning and decision-making. Through social media, students can access the latest information on job openings, career trends, and industry news. These platforms allow them to connect with potential employers, build professional networks, and stay informed about job opportunities. Research has consistently found that social media use is positively associated with students’ job choices by expanding their access to employment resources and enhancing their career awareness. Based on these findings, we propose that social media use is positively associated with college students’ job choices.

*Hypothesis 5*: Social media Use is positively associated with the job choices of college students.

**Figure 1:**
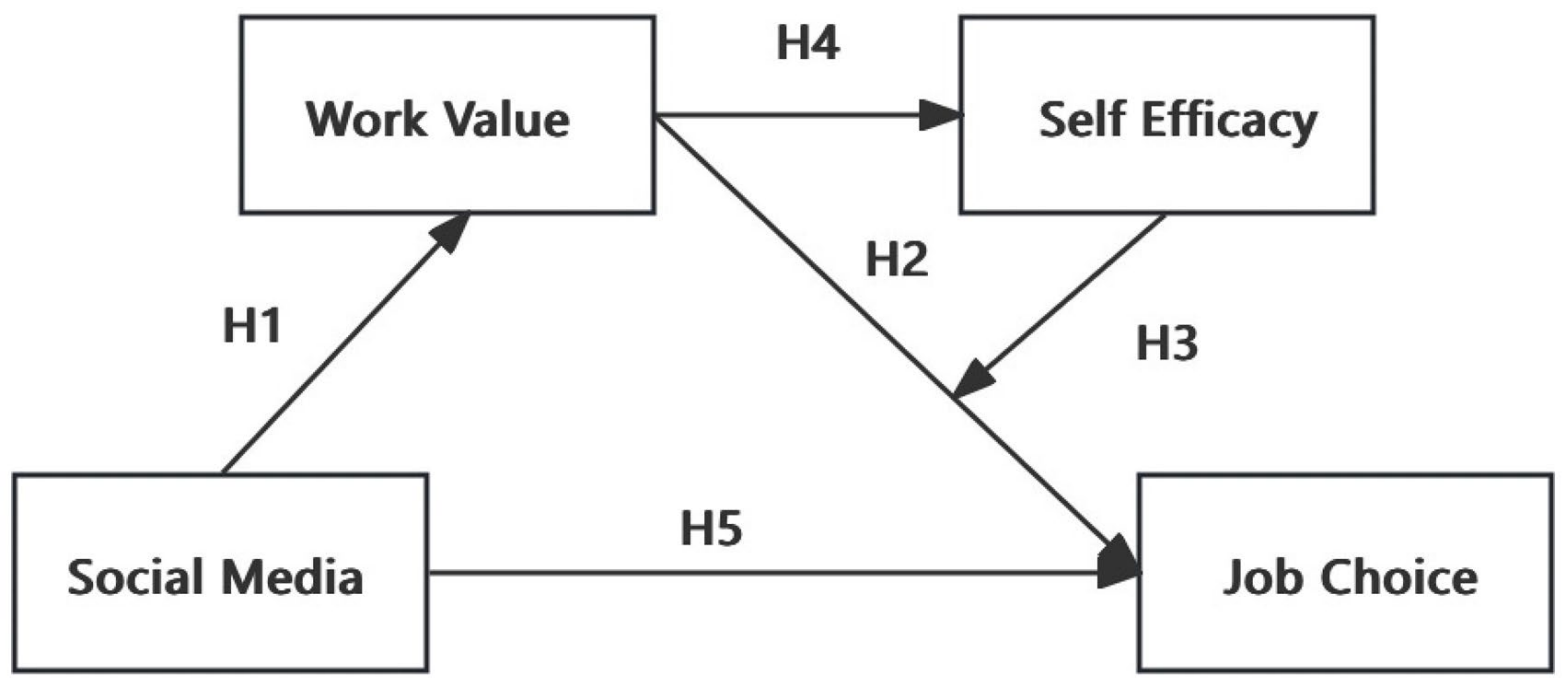
Hypothesized research model.

To summarize, this study added the factors of work values as well as self-efficacy on social media and job choice, and constructed a chain mediation model as shown in Figure 1.

## 4 Method

### 4.1 Participants

The sample for this study consisted of 254 college students from two universities in Anhui Province, selected through random sampling. The participants were aged 22.35 ± 2.14 years, with 105 male students and 149 female students. The sample size was determined based on *a priori* power analysis conducted using GPower 3.1, which recommended a minimum of 210 participants to achieve statistical validity (*α* = 0.05, power = 0.95). A total of 254 valid responses were obtained, resulting in a response rate of 98.7%.

### 4.2 Data collection

Data were collected through an online survey administered via the Question-Star platform. The survey was distributed in a classroom setting with the approval of the university’s teaching authority. The data collection process was carried out during students’ self-study sessions, allowing for approximately 15 min of survey time. Before completing the survey, students were informed of the study’s purpose, confidentiality, and the voluntary nature of their participation. The survey was designed to capture information on students’ social media use, work values, self-efficacy, and job choices.

### 4.3 Measures

Several scales were used to measure the key variables in this study.

Social media use: social media usage was measured based on three dimensions: frequency of use, duration of use, and level of interaction. A composite score was calculated using the formula:


Usage=frequency×duration×interaction level.

The scale had a maximum score of 100, and the Cronbach’s *α* for this scale was 0.88, indicating good reliability (Ozimek et al., 2023).

Work values: the work values scale consisted of eight items measuring six key dimensions: career fulfillment, job satisfaction, career development opportunities, work-life balance, work environment, and salary. Higher scores indicated greater importance placed on these dimensions when making career decisions. The Cronbach’s α for this scale was 0.83 (Avallone et al., 2010).

Job choice: job choice was measured using a scale consisting of 10 items, which covered six dimensions: career development opportunities, work environment, salary, career stability, work-life balance, and work interest. Higher scores indicated greater importance placed on these factors when choosing a job. The Cronbach’s *α* for this scale was 0.85 (Nadarajah et al., 2022).

Self-efficacy: the self-efficacy scale included 10 items across six dimensions: self-esteem, self-confidence, personal growth, social support, emotional regulation, and goal setting. Higher scores on this scale reflected stronger self-efficacy. The Cronbach’s *α* for this scale was 0.86 (Bandura, 2006).

### 4.4 Data analysis

The data were analyzed using multiple regression and bootstrap methods to test the mediation effects of work values and self-efficacy. Specifically, we tested the association between social media use and job choices, considering the role of work values and self-efficacy as mediators. To ensure robust results, a bootstrapping method was used to verify the significance of indirect associations. The analysis was conducted using SPSS and PROCESS macro to estimate the mediation effects and test the hypotheses proposed in this study.

Through this analysis, we aim to explore the potential associations between social media usage and college students’ employment choices, examining the intricate relationships involving work values and self-efficacy. The findings will contribute to the literature on social media use and job choices, and provide practical implications for career guidance and policy-making (Wang and Yang, 2014).

## 5 Results

### 5.1 Descriptive statistics

Correlation analysis showed (Table 1) Social media use is significantly and positively associated with work values among college students (=0.175, *p* < 0.01) and with self-efficacy (*r* = 0.210, *p* < 0.01). Work values (defined operationally as beliefs and priorities such as job stability, career advancement, and work-life balance) are significantly and positively related to self-efficacy (=0.198, *p* < 0.01). As the frequency of social media use increased among college students, their work values and sense of self-empowerment also showed a corresponding increased. In addition, work values were significantly and positively related to employment choices (*r* = 0.322, *p* < 0.01) and significantly positively correlated with self-efficacy (=0.289, *p* < 0.01). When individuals’ work values are higher, they are more inclined to value personal development and career fulfilment in their employment choices. Meanwhile, when individuals’ sense of self-efficacy increases, they are more confident and decision-making when making employment choices.

**Table 1:** The mean value, standard deviation and relevant statistical results of each variable.

Variable	*M*	SD	1	2	3
Social media	35.42	12.85			
Employment choices	82.56	14.78	0.175**		
Self-efficacy	45.32	10.24	0.210**	0.198**	
Work values	60.14	11.52	0.322**	0.289**	0.276**

* indicates *p* < 0.05, ** indicates *P* < 0.01, *N* = 254.

### 5.2 The association between social media use and job choices: a regression analysis of work values and self-efficacy

In order to explore the associations between social media use, work values, and self-efficacy with employment choice, on the basis of correlation analysis, regression analysis was conducted with the former three as independent variables and employment choice as the dependent variable. The specific statistical results are shown in Table 2. As can be seen from Table 2, the independent variable that enters into the regression equation in Model 1 is social media use, and there is a significant positive association of social media use with employment choice (*β* = 0.322, *t* = 5.112, *p* < 0.01), and its explained amount is 10.4%; after the inclusion of word values into the regression equation in model 2, there is a significant positive association of work values with employment choice (*β* = 0.289, *t* = 6.253, *p* < 0.01), and the joint explained variance (*R*^2^) of social media use and work values is 16.5%, of which The amount of explained variance (∆*R*^2^) of work values is 6.1%; after self-efficacy is again included in the regression equation in model 3, there is a significant positive association of self-efficacy with employment choice (*β* = 0.372, *t* = 7.512, *p* < 0.01), and the amount of jointly explained variance (*R*^2^) of the three variables on employment choice is 28.4%, of which, the amount of explained variance (∆*R*^2^) of self-efficacy is 11.9%, and the individual explanations added by each independent variable into the regression model reached significance. These results suggests that social media use may be associated with employment choice through work values and self-efficacy.

**Table 2:** The regression analysis results of social media use, work values, and self-efficacy on employment choices.

Independent variables	Employment choices β(1)	*t*(1)	Employment choices β(2)	*t*(2)	Employment choices β(3)	*t*(3)
Social media use	0.322	5.112**	0.118	3.989**	–	–
Work values	–	–	0.289	6.253**	−0.259	8.851**
Self-efficacy	–	–	–	–	0.372	7.512**
*R* ^2^	0.104		0.165		0.284	
△*R*^2^	10.104		0.061		0.119	
*F*	26.145**		45.372**		33.071**	

* indicates *P* < 0.05, ** indicates *P* < 0.01, *N* = 254.

### 5.3 Intermediation analysis

Based on the mediation effect testing process proposed by Zhonglin and Baojuan (2014). In the first step, the coefficient *c* of social media use on employment choice was tested (*c* = 0.322, *t* = 5.112, *p* < 0.01), and the mediation effect between social media use and employment choice was established. In the second step, the coefficients a1 = 0.175, a2 = 0.210; coefficients b1 = 0.289, b2 = 0.372 were tested sequentially; all four coefficients were significant and the indirect effect was significant. In the third step, in order to examine the chain mediation effect (reorganized for clarity as described in the discussion) of work values and self-efficacy, the Bootstrap method was used to conduct the chain mediation effect (reorganized for clarity as described in the discussion) test, the test method model selection 6, the sample size selection 5,000, to see whether the mediation effect is significant under the 95% confidence interval, and the test results show that (Table 3): (1) The mediating effect value for work values was 0.0152, and its 95% CI is [0.0100, 0.0204]; (2) the mediation effect size of self-efficacy was 0.0346, with a 95% CI of [0.0220, 0.0472]; and (3) the chained mediation effect value of the two was 0.0024, with a 95% CI of [0.0010, 0.0038]. The intervals of the above three paths do not include 0, indicating that each mediating effect is significant. Further decomposing the effect size of each variable on employment choice, the direct effect of social media use to employment choice was 0.0721, the total indirect effect value of 0.0231 was the sum of the mediating effects of the 3 mediating paths, and the sum of the direct effect and the total indirect effect was the total effect of 0.0952. The value of each mediating effect was divided by the total effect as the effect size. The effect shares of the three mediating paths in this paper are 15.97, 36.37, and 2.52%, respectively.

**Table 3:** Results of intermediary analyses.

Influence path	Effect	Boot standard error	Boot 95%Cl lower	Boot 95%Cl upper	Relative mediated effect
Ind1	0.0152	0.0030	0.0100	0.0204	15.97%
Ind2	0.0346	0.0055	0.0220	0.0472	36.37%
Ind3	0.0024	0.0014	0.0010	0.0038	2.52%
Total indirect effect	0.0231	0.0087	0.0120	0.0342	54.86%

The study shows that when social media use changes by one standard deviation, employment choice will change by 0.0952 standard deviations. Among them, 0.0231 standard deviations were generated by indirect mediating effects, the mediating effect of work values produced a facilitating effect, which accounted for 15.97% of the total indirect effect, while the mediating effect of self-efficacy played a positive facilitating effect, which accounted for 36.37% of the total effect, and the interaction between the two also played a positive facilitating effect, which accounted for 2.52% of the total effect. Comparing the mediating effects of the three different paths, the results showed that, as far as the sole mediating effect was concerned, the positive effect of self-efficacy was significantly higher than that of work values (*E* = 0.0194, LLCI = 0.0120, ULCI = 0.0268). Their chained mediation effects were all lower than their respective stand-alone mediation effects, and there was a significant difference between them and the mediation effect of work values (*E* = −0.0076, LLCI = −0.0120, ULCI = −0.0032), and a significant difference between them and the mediation effect of self-efficacy (*E* = 0.0122, LLCI = 0.0064, ULCI = 0.0180).

## 6 Discussion and conclusion

The purpose of this study was to explore the associations between social media use and college students’ career choices and to analyze the roles of work values and self-efficacy through a chain mediation model. Based on the data analysis of 254 college students, regression analysis and Bootstrap test revealed several important findings. These results highlight the potential associations between social media use and career choice, and they underscore the relationships of work values and self-efficacy as important factors in this process.

### 6.1 Direct association of social media use with job choice

The study found that there is a significant positive correlation between the frequency of social media use and the job choice of college students. Specifically, the coefficient of the direct association was 0.322, with a t-value of 5.112 and a *p*-value of less than 0.001. This suggests that increased social media use is positively associated with flexibility and diversity in job choice. Hypotheses 1 and 5 were supported, and this result is consistent with the existing literature, emphasizing the important contribution of social media in the job decision-making process of contemporary college students. Social media provides students with a wealth of information and resources to better understand various job opportunities and their requirements. By interacting with career experts, alumni, and peers, students can gain valuable career guidance and advice (Austin, 2002). In addition, social media provides a platform for students to showcase their abilities and achievements, enhancing their competitiveness in the job market. These factors work together to make students with higher frequency of social media use show greater diversity and flexibility in their job choices.

### 6.2 Mediating effects of work values and self-efficacy

In addition to the direct association, the study found that the relationship between social media and job choice was further explained through the mediating role of work values and self-efficacy. In the chain mediation model, the total contribution of social media use on job choice was 0.0952, of which the direct effect was 0.0721 and the indirect effect was 0.0231, and both were statistically significant. Specifically, social media is related to job choice indirectly by enhancing work values and self-efficacy. The mediating effect of work values was 0.0152, accounting for 15.97% of the total effect; the mediating effect of self-efficacy was 0.0346, accounting for 36.37%; and the interaction effect of work values and self-efficacy was 0.0024, accounting for 2.52%. These results suggest that social media not only affects job choice directly but also works indirectly by influencing students’ work values and self-efficacy.

Work values (which in this study refers to beliefs and priorities about job stability, career development opportunities, and work-life balance) reflect an individual’s understanding of the meaning and importance of work, which in turn directly associates with his or her job choices and occupational behaviors. Social media enables students to be exposed to a diversity of work values and role models, thus helping them to form and strengthen their personal work values (Tess, 2013). On the other hand, self-efficacy is the process of increasing self-confidence through positive self-evaluation, and social media, as a platform for displaying personal accomplishments, helps to enhance students’ self-identity, enabling them to show more confidence and determination in their job choices.

In order to verify the chain mediation effect of work values and self-efficacy, this study used the Bootstrap method with a sample size of 5,000 and a significance test with a 95% confidence interval. The results showed that the mediating effect of work values was 0.0152 with a 95% confidence interval of [0.0100, 0.0204]; the mediating effect of self-efficacy was 0.0346 with a 95% confidence interval of [0.0220, 0.0472]; and the interaction effect of work values and self-efficacy was 0.0024 with a 95% confidence interval of [0.0010, 0.0038]. Since none of these confidence intervals contained zero, all mediating effects were statistically significant. Thus, hypotheses 2, 3, and 4 were supported, further validating the indirect association of social media use with job choice and the critical role of work values and self-efficacy in this process.

### 6.3 Conclusion

The findings of this study offer valuable theoretical and practical insights. Theoretically, it enhances the understanding of the associations between social media use, work values, self-efficacy, and job choice, revealing the complex interrelationships among these variables (Hartman and Barber, 2020). This research expands the theoretical framework on the role of social media in career decision-making and offers new directions for future studies.

Practically, the findings provide guidance for educators, career counselors, and universities. Educators should incorporate social media literacy into curricula to help students navigate reliable career information. Career counselors should focus on strengthening students’ work values and self-efficacy, enabling more informed and confident job decisions (Falco et al., 2019). Universities should leverage social media for career awareness and networking, while teaching students to critically assess online information to ensure well-informed choices.

In conclusion, this study underscores the critical relevance of social media use, work values, and self-efficacy on college students’ job choices, suggesting that vocational education should consider the impact of social media. Future research can further explore these mechanisms across diverse groups and cultural contexts, providing deeper insights into the role of digital platforms in modern career development.

## 7 Limitation and future research

Despite some valuable results from this study, there are some limitations. First, this study used cross-sectional data, which did not allow for the identification of causal relationships. Future research could use a longitudinal design to further validate the causal relationship between social media use, work values, self-efficacy, and employment choices. Second, the sample of this study was from college students in a specific region, and the generalizability of the results may be limited. Future research could expand the sample to verify the generalizability of the results. Future studies can also explore other potential mediating variables, such as social support and career adaptability, to further enrich the theoretical framework of the impact of social media use on employment choice. In addition, the study can also focus on the different effects of different types of social media (e.g., professional social media, entertainment social media) on employment choice, to gain a deeper understanding of the mechanism of social media’s role in career decision-making.

## Data Availability

The original contributions presented in the study are included in the article/supplementary material, further inquiries can be directed to the corresponding author.
